# Supplementary material for: Phalloidin and DNase I-bound F-actin pointed end structures reveal principles of filament stabilization and disassembly
Source: Nat Commun. 2024 Sep 11;15:7969. doi: 10.1038/s41467-024-52251-3 (PMC11390976; doi:10.1038/s41467-024-52251-3)
Supplement: Supplementary file 1 — Supplementary Information [file 41467_2024_52251_MOESM1_ESM.pdf]

Supplementary information for:

Phalloidin and DNase I-bound F-actin pointed end structures reveal principles of filament stabilization and disassembly

Micaela Boiero Sanders[1§], Wout Oosterheert[1§], Oliver Hofnagel[1], Peter Bieling[2*], Stefan Raunser[1*]

[1]Department of Structural Biochemistry, Max Planck Institute of Molecular Physiology, 44227 Dortmund, Germany

[2]Department of Systemic Cell Biology, Max Planck Institute of Molecular Physiology, 44227 Dortmund, Germany

[§]These authors contributed equally: Micaela Boiero Sanders and Wout Oosterheert

*Correspondence to:
Peter Bieling, peter.bieling@mpi-dortmund.mpg.de
Stefan Raunser, stefan.raunser@mpi-dortmund.mpg.de

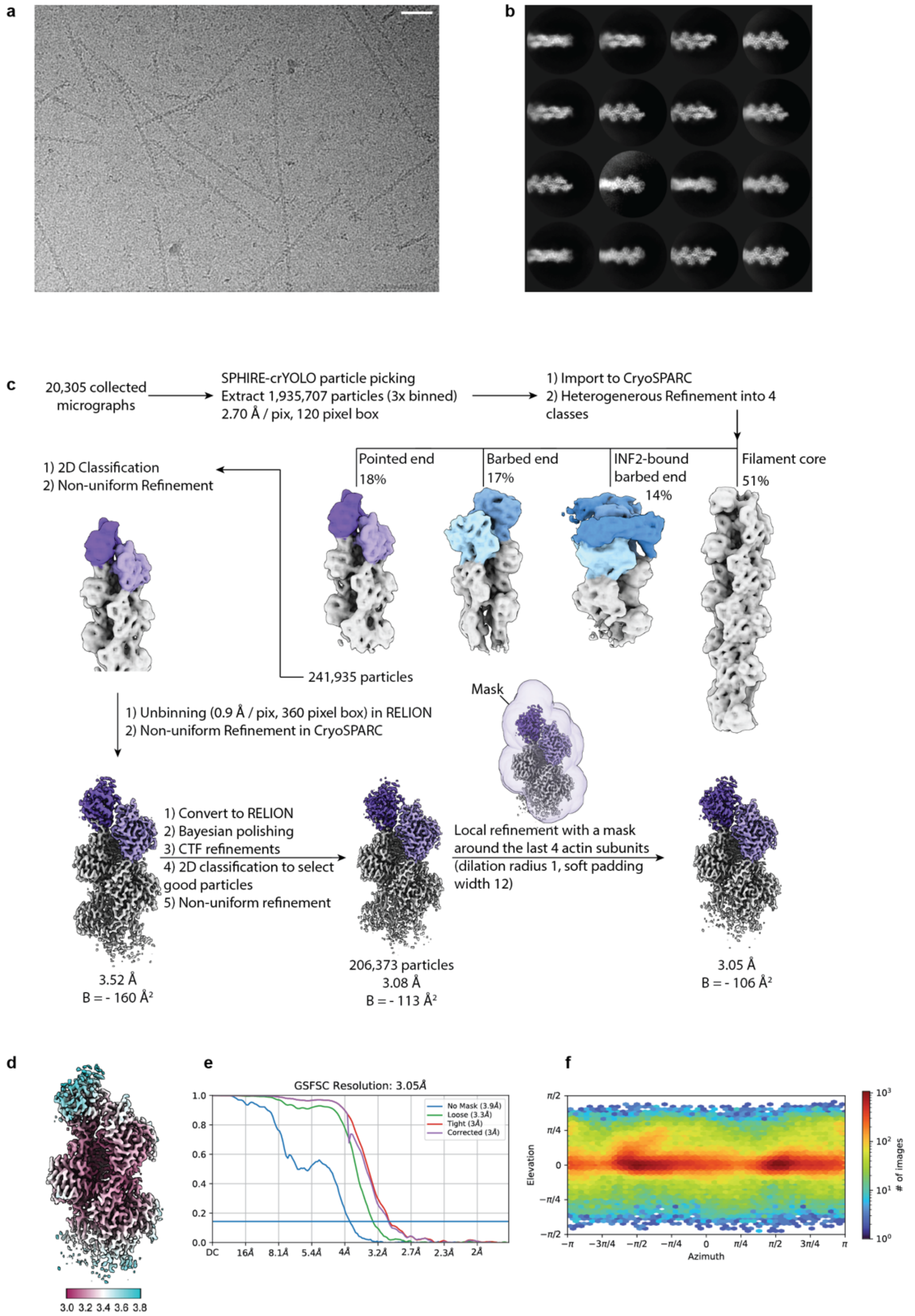

Supplementary Figure 1. Cryo-EM image processing workflow for the undecorated pointed end of actin filaments.

a Representative micrograph showing short actin filaments polymerized and severed by INF2, at a defocus of -2.5 µm, from a dataset comprising a total of 20,305 micrographs. Scale bar: 400 Å. **b** Selected 2D classes of the undecorated F-actin pointed end. Box size is 324 x 324 Å. **c** Image processing strategy used to obtain the density map corresponding to the undecorated

pointed end. The last two subunits of the pointed end and the last two subunits of the barbed end are colored purple and light blue, respectively. The formin INF2 is colored blue. **d** Local-resolution estimations of the undecorated pointed end density map, calculated by CryoSPARC. The bar shows the color palette used to color the density map, and the corresponding resolution in Å. **e** Fourier-shell correlation plots for the undecorated pointed end reconstruction, calculated by CryoSPARC. The curves were calculated after applying a loose mask (green), a tight mask (red), no mask (light blue) or a tight mask and correction by noise substitution (purple). **f** Angular distribution of the particles used for the reconstruction of the final map of the pointed end, calculated by CryoSPARC.

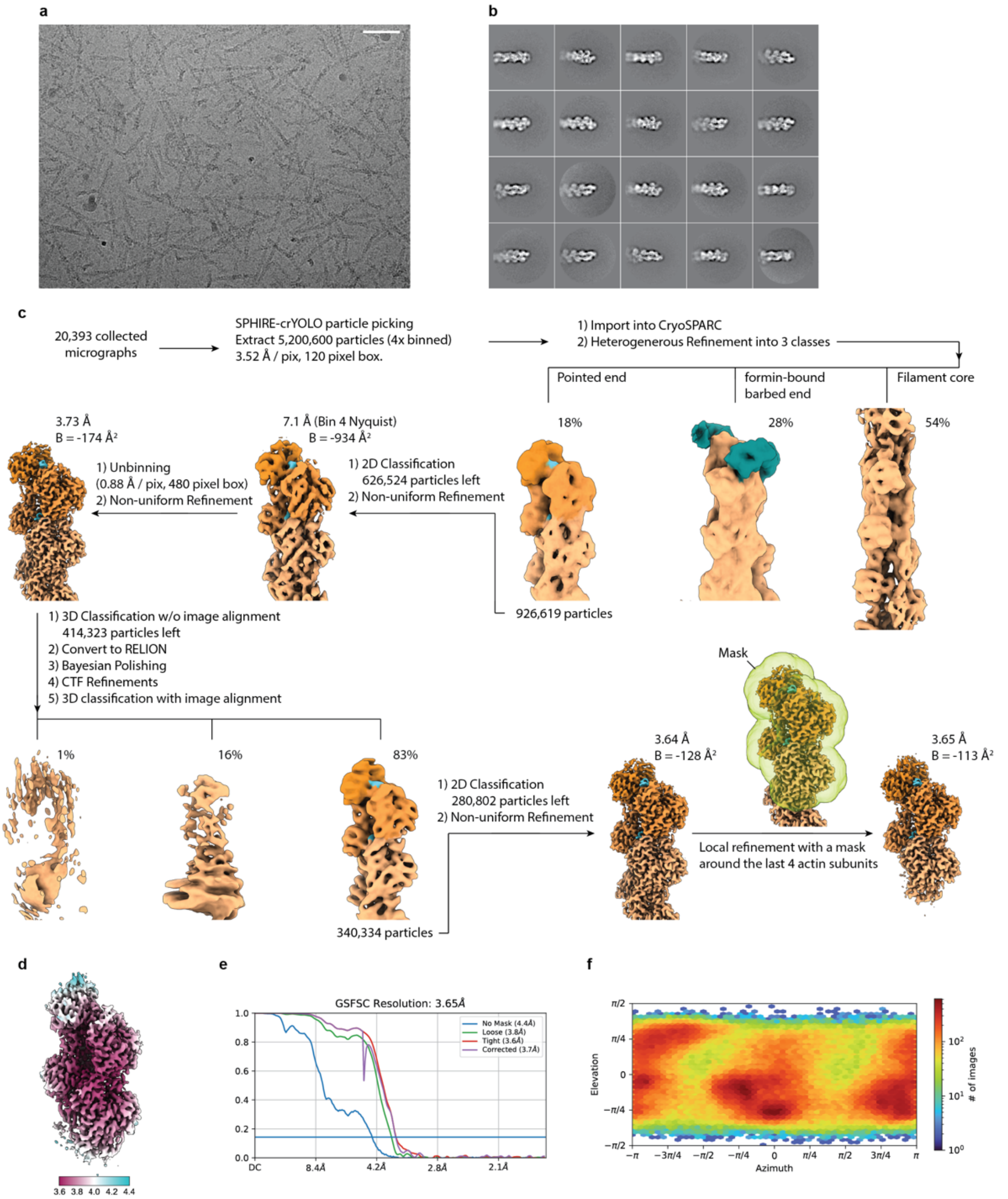

Supplementary Figure 2. Cryo-EM image processing workflow for the phalloidin-bound pointed end of F-actin.

a Representative micrograph showing short actin filaments polymerized in the presence of Cdc12, at a defocus of -1.9 µm, from a dataset comprising a total of 20,393 micrographs. Scale bar: 400 Å. **b** Selected 2D classes of the phalloidin-bound F-actin pointed end. Box size is 422 x 422 Å. **c** Image processing strategy used to obtain the density map corresponding to the phalloidin-bound pointed end. Phalloidin-bound actin subunits are colored in light orange, and the last two subunits of the pointed end are colored in darker shades of orange, phalloidin is colored cyan and Cdc12 is colored teal. **d** Local-resolution estimations of the phalloidin-bound pointed end density map, calculated by CryoSPARC. The bar shows the color palette used to

color the density map, and the corresponding resolution in Å. **e** Fourier-shell correlation plots for the phalloidin-bound pointed end reconstruction, calculated by CryoSPARC. The curves were computed after applying a loose mask (green), a tight mask (red), no mask (light blue) or a tight mask and correction by noise substitution (purple). **f** Angular distribution of the particles used for the reconstruction of the final phalloidin-bound pointed end map, calculated by CryoSPARC.

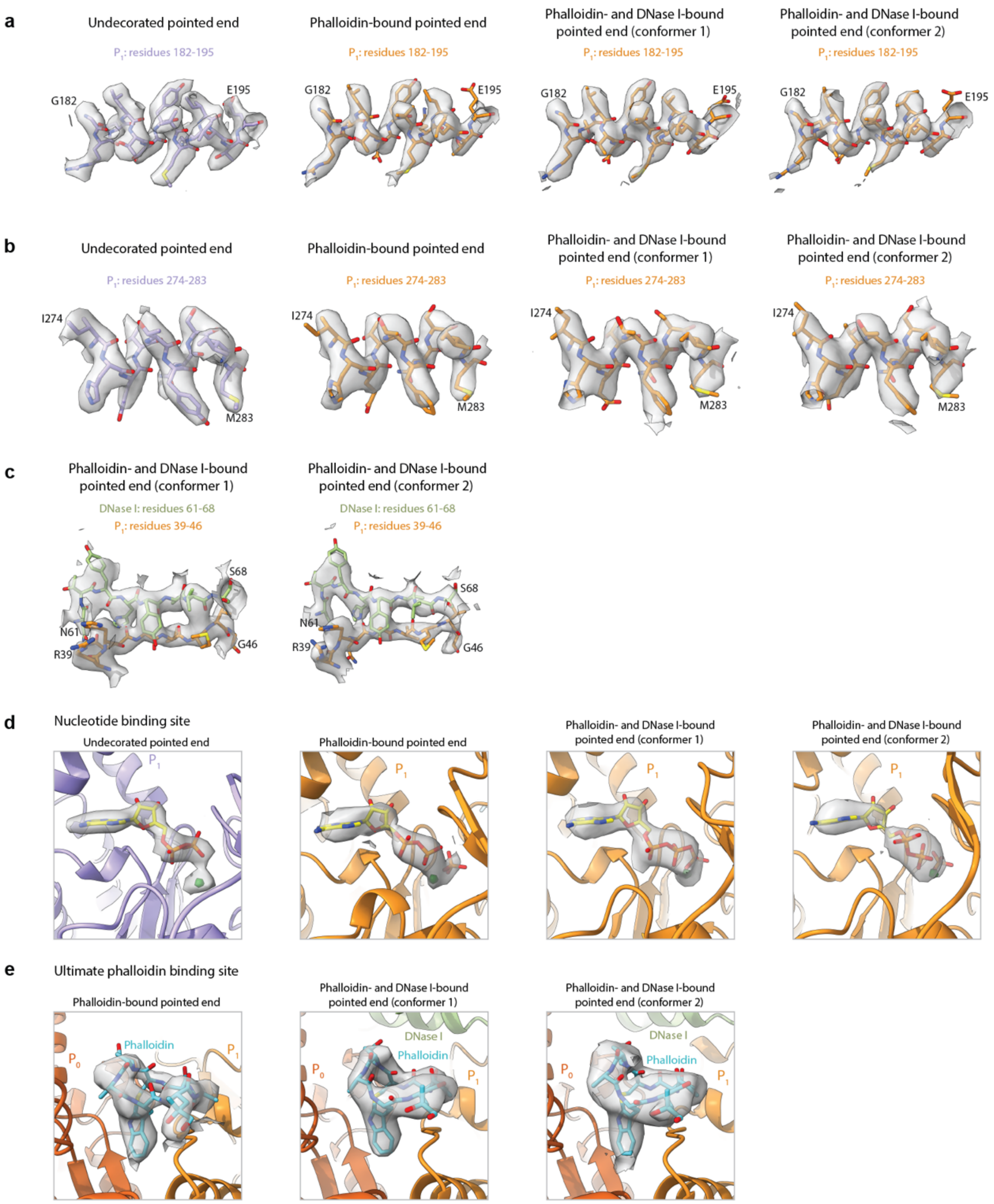

Supplementary Figure 3. Local Densities of selected regions.

Overlay of selected regions of the cryo-EM maps and modeled structures for all structures presented in this article (undecorated pointed end, phalloidin-bound pointed end and the two conformers of the phalloidin- and DNase I bound pointed end). **a** Cryo-EM local densities of residues G182 – E195 for the penultimate actin subunit P_1 of all structures. **b** Cryo-EM local densities of residues I274 – M283 for actin subunit P_1 of all structures. **c** Cryo-EM local densities of DNase I residues N61 – S68 and actin subunit P_1 residues R39 – G46 for both conformers of the phalloidin- and DNase I-bound pointed end. **d, e** Cryo-EM local densities of the nucleotide (d) and phalloidin (e) binding sites.

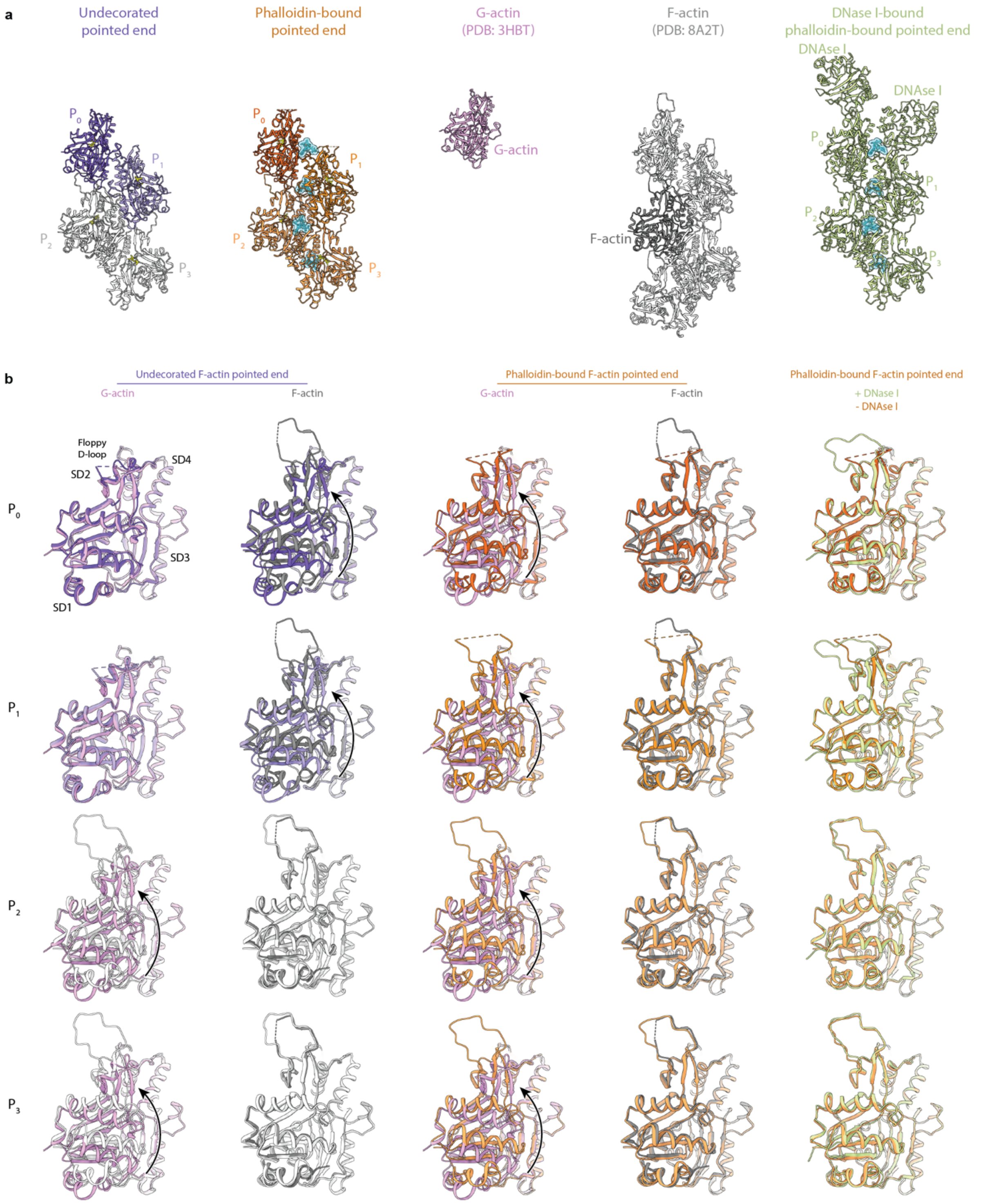

Supplementary Figure 4. Comparison of the actin subunits of the pointed end structures. **a** Structures of all maps used for this comparison: undecorated pointed end (purple, light grey, this study), phalloidin-bound pointed end (orange, this study), G-actin (pink, PDB: 3HBT), F-actin (light/dark grey, PDB: 8A2T) and the phalloidin-bound, DNase I-decorated pointed end (light green, this study). **b** The most terminal 4 actin subunits (P_0-P_3) of the undecorated pointed end (purple and light grey, first two columns) and the phalloidin-bound pointed end (orange,

third and fourth column) were compared against a typical unflattened actin monomer (G-actin, pink, PDB: 3HBT) and against a typical filamentous actin subunit (F-actin, dark grey, PDB: 8A2T). In the last column, the subunits of the phalloidin-bound pointed end (orange) were compared against the subunits of the phalloidin-bound, DNase I-decorated pointed end (light green). Arrows highlight where we can observe the typical 12.4° rotation of the SD1 and SD2 between flattened and unflattened actin subunits.

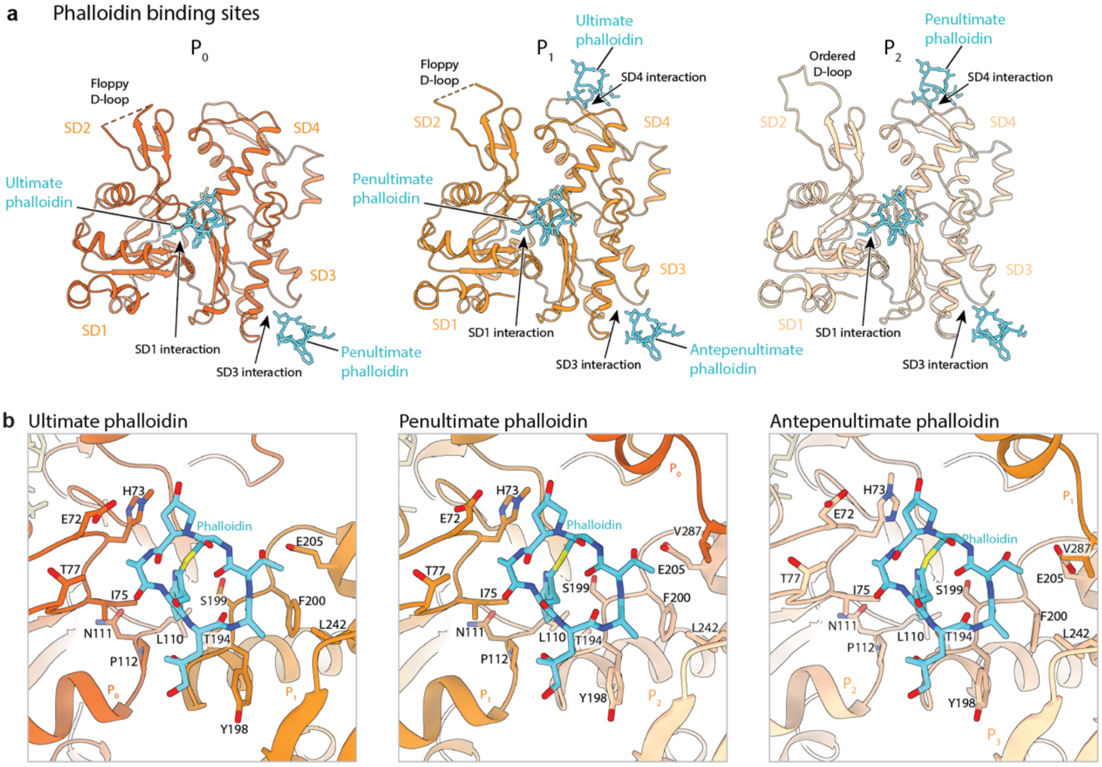

Supplementary Figure 5. Phalloidin molecules interacting with the last 3 subunits of the pointed end.

a Illustration of all phalloidin (light blue) interactions for the three terminal subunits of the pointed end P_0 (red-orange), P_1 (orange) and P_2 (light orange). **b** Zoom-in images of the molecular interaction between the ultimate phalloidin (left panel), the penultimate phalloidin (mid panel) and the antepenultimate phalloidin (right panel) with the different actin subunits at the pointed end of F-actin.

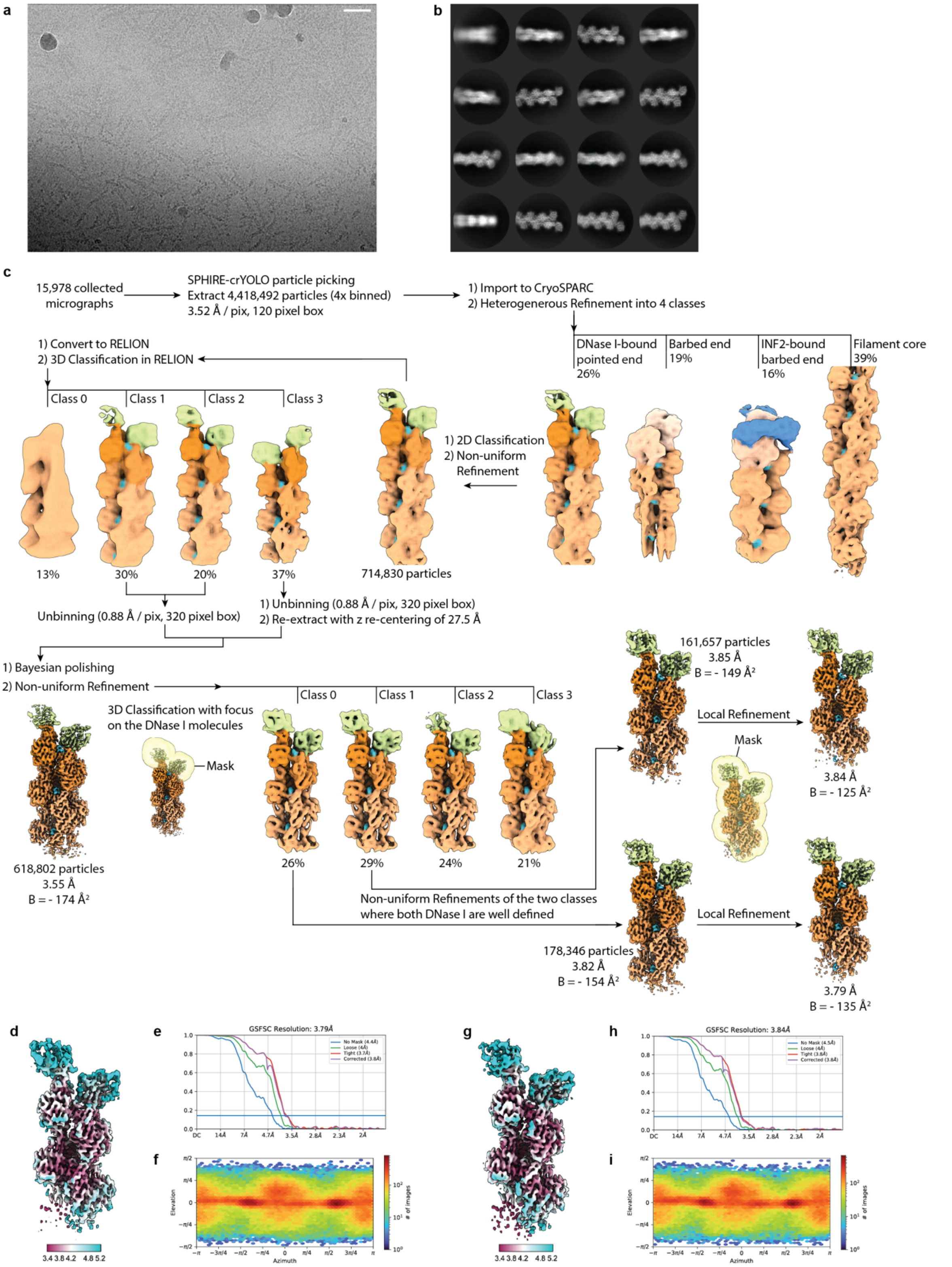

Supplementary Figure 6. Cryo-EM image processing workflow for the DNase I-bound pointed end of F-actin.

a Representative micrograph showing short actin filaments polymerized in the presence of phalloidin, DNase I and INF2, at a defocus of -2.3 µm, from a dataset comprising a total of 15,977 micrographs. Scale bar: 400 Å. **b** Selected 2D classes of the phalloidin and DNase I-

bound F-actin pointed end. Box size is 282 x 282 Å. **c** Imaging processing strategy used to obtain the density maps corresponding to the phalloidin and DNase I-bound pointed end. Phalloidin-bound actin subunits are colored in light orange, and the last two subunits of the pointed end are colored in darker shades of orange, phalloidin is colored cyan, INF2 is colored in blue and DNase I in light green. **d, g** Local-resolution estimations of the phalloidin and DNase I-bound pointed end density maps, calculated by CryoSPARC. The bar shows the color palette used to color the density map, and the corresponding resolution in Å. **e, h** Fourier-shell correlation plots for the phalloidin and DNase I-bound pointed end reconstruction, calculated by CryoSPARC. The curves were computed after applying a loose mask (green), a tight mask (red), no mask (light blue) or a tight mask and correction by noise substitution (purple). **f, i** Angular distribution of the particles used for the reconstruction of the final phalloidin and DNase I-bound pointed end maps, calculated by CryoSPARC.

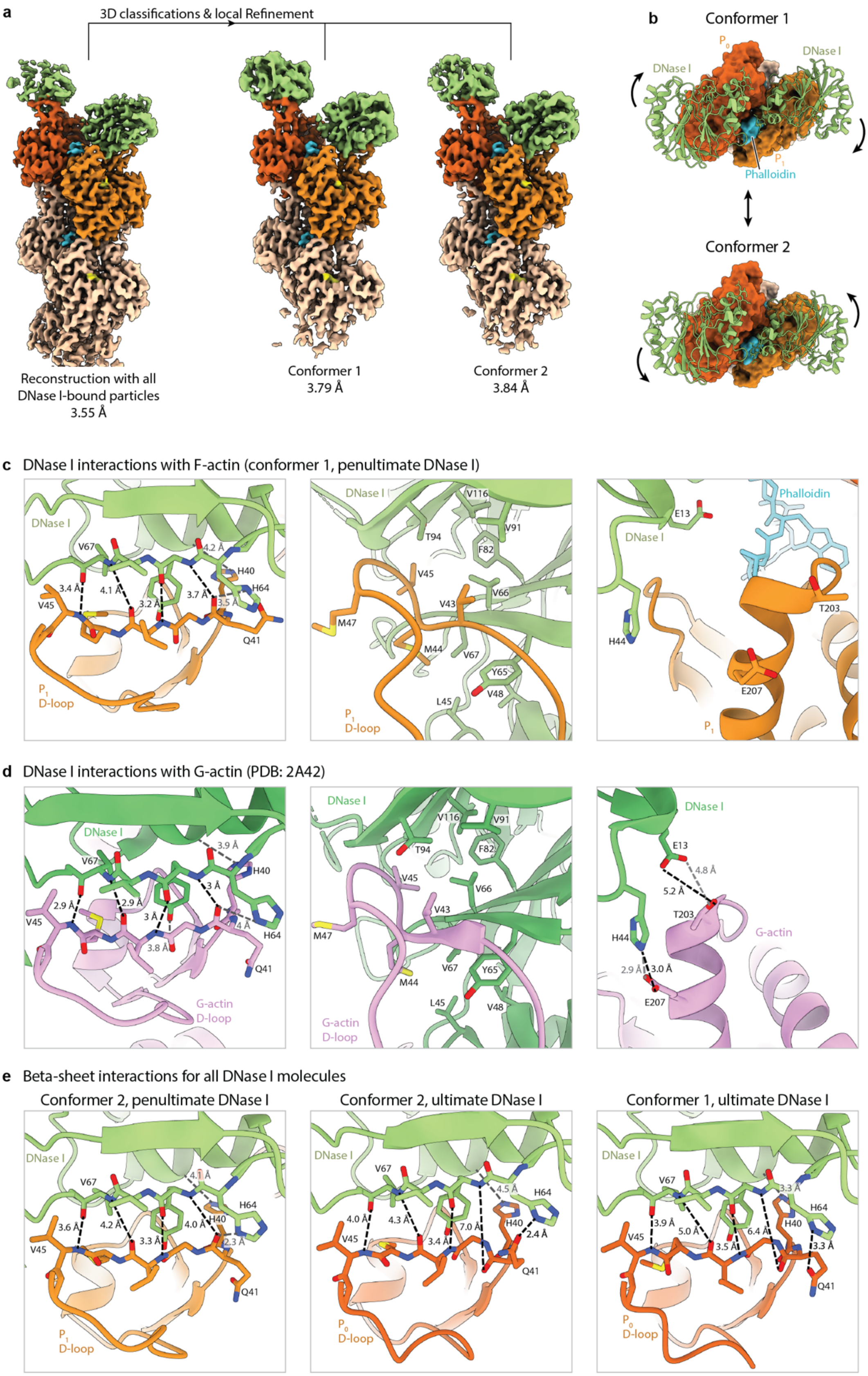

Supplementary Figure 7. Comparison of the DNase I binding sites in F-actin and G-actin. **a** Cryo-EM density maps of the DNase I- and phalloidin-bound pointed end of F-actin which was reconstructed with all DNase I-bound pointed end particles (left). Cryo-EM density maps

of the two conformers obtained after 3D classification (middle and right). Phalloidin-bound actin subunits are colored from dark orange at the pointed end to lighter orange towards the filament center. The two bound DNase I molecules, the bound nucleotide and phalloidin are colored light green, yellow and cyan, respectively. **b** Top view of the structure of conformers 1 and 2 (modeled after maps obtained by 3D classification), see also the 3D variability results in Supplementary Video 1. **c** Zoom-in images of the molecular interaction between the penultimate DNase I molecule (light green) and the penultimate actin subunit of the phalloidin-bound pointed end (P_1, orange). Beta sheet-like interactions (left), hydrophobic interactions (middle) and a lost interaction (right) are shown. The left and middle panels were taken from Fig. 3d and 3e, respectively. **d** Zoom-in images of the molecular interaction between DNase I (dark green) and monomeric actin (G-actin, pink). Beta sheet-like interactions (left), hydrophobic interactions (middle) and a unique DNase I G-actin interaction (right) are shown. **e** Beta sheet-like interactions between DNase I and actin, showed for all remaining DNase I molecules from both conformers.

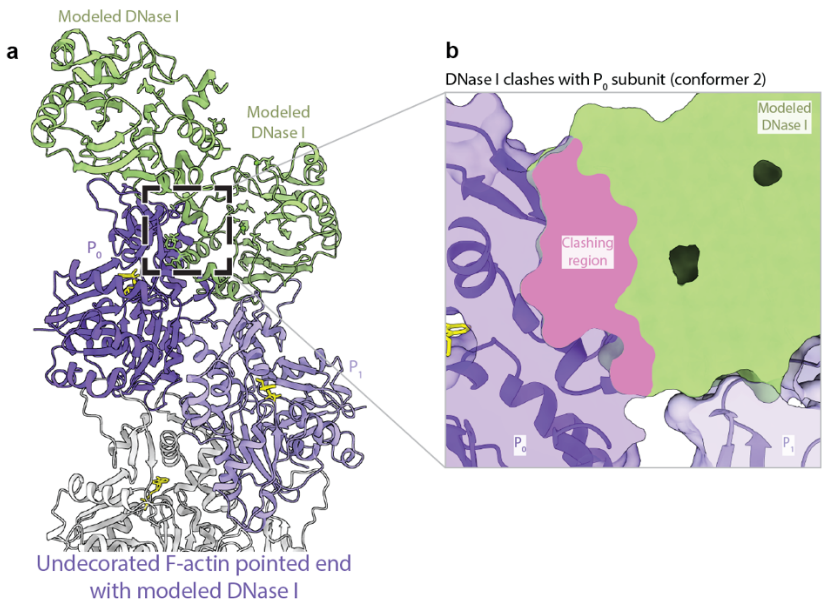

Supplementary Figure 8. Both conformers of DNase I would displace P_0 in the native pointed end.

a Alignment of the DNase I-bound pointed end with the undecorated pointed end, for conformer 2 (see Fig. 4 for conformer 1). Actin subunits are labeled depending on their location on the filament, so that the ultimate subunit is P_0 and subunits towards the filament center have an increasing number (P_1, P_2, P_3). Actin subunits are colored in purple and grey, whereas DNase I is colored light green. The bound nucleotide is highlighted in yellow. **b** Zoom-in image of the clashes between the penultimate DNase I and the actin subunit P_0. The clashing region is shown in pink.

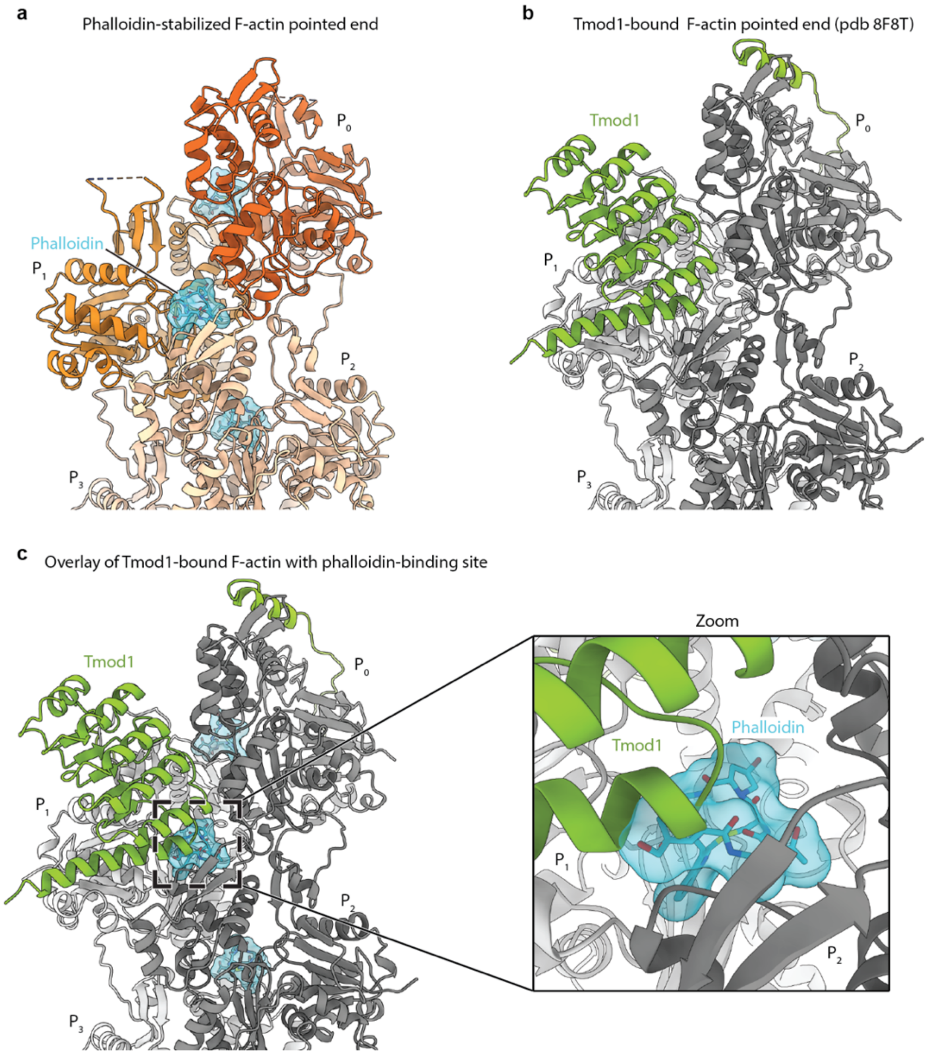

Supplementary Figure 9. Modeled phalloidin binding site in the tropomodulin-bound F-actin structure.

a Structure of the phalloidin-bound pointed end. Actin subunits are shown in orange, phalloidin is colored cyan. **b** Structure of the tropomodulin 1 (Tmod1)-bound pointed end (PDB: 8F8T). Tmod1 is colored green, while the two strands of actin are colored in dark and light grey. **c** Overlay of the Tmod1-bound pointed end structure (green for Tmod1, light and dark grey for actin) and phalloidin (cyan) (right). Zoomed-in image of the penultimate phalloidin binding site, showing the clashes between phalloidin and Tmod1, indicating that the binding of both molecules to the pointed end is not possible without structural rearrangements (left). For all maps, actin subunits are labeled with suffixes of increasing numbers starting from the terminal actin subunit of the pointed end (P_0) towards the filament center (P_3).

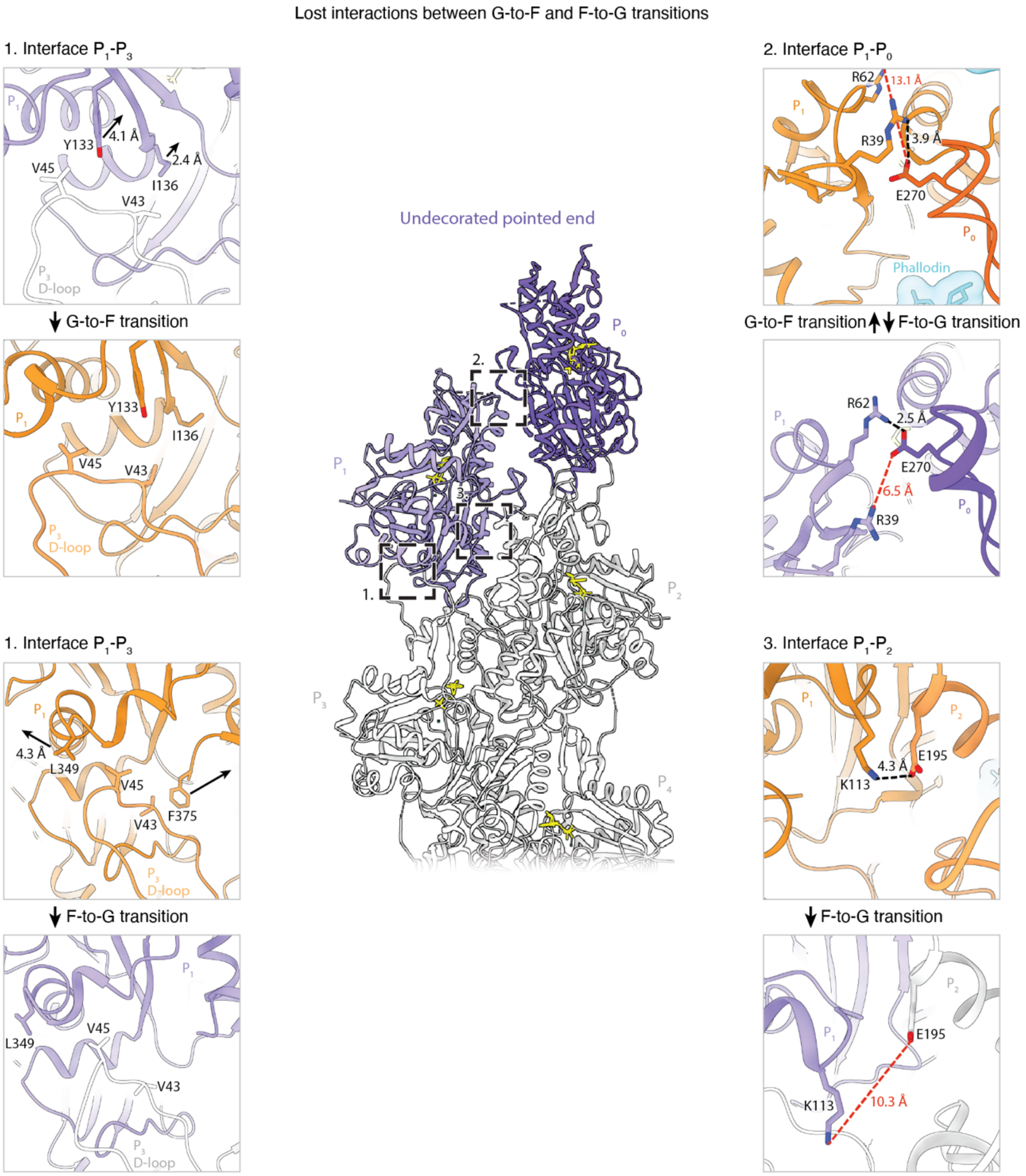

Supplementary Figure 10. Lost interactions during G-to-F and F-to-G transitions of P_1. (Center) Model of the undecorated pointed end of actin filaments, with the ultimate subunit (P_0) colored in purple, the penultimate subunit (P_1) colored in lilac, and subsequent subunits towards the filament center (P_2 and beyond) colored in light grey. The interfaces of P_1 with the neighboring subunits are highlighted with dashed-line squares: 1. Interaction between SD1 of P_1 and the D-loop of P_3; 2. interaction between SD2 of P_1 and the H-plug (SD4) of P_0; 3. interaction between the proline rich loop of P_1 and SD4 of P_2. (Left side) Zoom-in on the rearrangements between P_1 and P_3 (interface 1) during flattening (G-to-F) and twisting (F-to-G) of P_1. The structure in orange corresponds to the phalloidin bound pointed end, as an example of the arrangement of the same region when the subunits are in a flattened

conformation. Exemplary amino acids that change positions during this transition are annotated: P_1-Y133 and P_1-I136 interactions with the D-loop are disrupted during flattening, while P_1-L349 and the C-terminal P1-F375 interactions with the D-loop are disrupted during twisting. (Right side) Zoom-in on the rearrangements between P_1 and P_0 (interface 2), as well as the interactions between P_1 and P_2 (interface 3) during flattening and unflattening of P_1. The structure in orange corresponds to the phalloidin bound pointed end, as an example of the arrangement of the same region when the subunits are in a flattened conformation. Exemplary amino acids that change positions during this transition are annotated: For interface 2 the salt bridge between P_1-E270 and P_2-R39 needs to break during twisting to form a new salt bridge between P_1-E270 and P_2-R62 (and vice versa during flattening); for interface 3 the salt bridge between P_2-K113 and P_3-E195 is disrupted during twisting. All these changes in the interaction interfaces possibly create a potential barrier for P_1 to undergo a conformational change.

Supplementary Table 1. Cryo-EM data collection, refinement and validation statistics.

dataset	α-actin pointed end, undecorated<br><br>EMD-50507<br>PDB 9FJO	β-actin pointed end, phalloidin-bound<br><br>EMD-50506<br>PDB 9FJM
**Data collection and processing**		
Magnification	105,000	81,000
Voltage (kV)	300	300
Electron exposure (e–/Å^2)	60.6	64.6
Nominal defocus range (μm)	-1.3 to -2.9	-1.2 to -2.7
Pixel size (Å)	0.9	0.88
Symmetry imposed	C1	C1
Initial particle images (no.)	1,935,707	5,200,600
Final particle images (no.)	206,373	280,802
Map resolution (Å)	3.05	3.65
0.143 FSC threshold		
Map resolution range (Å)	2.9 – 3.9	3.6 – 4.4
**Refinement**		
Initial model used (PDB code)	8A2T	8RTT
Model resolution (Å)		
0.5 FSC threshold	3.3	3.8
Map sharpening *B* factor (Å^2)	-106	-113
Model composition		
Non-hydrogen atoms	11,466	11,772
Protein residues	1,452	1,487
Waters	0	0
Ligands	8	16
B factors (Å^2)		
Protein	68.93	57.36
Waters	-	-
Ligand	49.53	55.09
R.m.s. deviations		
Bond lengths (Å)	0.002	0.002
Bond angles (°)	0.523	0.573
Validation		
EM-ringer score	3.73	2.24
MolProbity score	1.33	1.45
Clashscore	5.36	8.19
Rotamer outliers (%)	0.24	0.16
Ramachandran plot		
Favored (%)	97.83	98.54
Allowed (%)	2.17	1.46
Disallowed (%)	0.00	0.00

Supplementary Table 2. Cryo-EM data collection, refinement and validation statistics.

dataset	**β/γ-actin pointed end, phalloidin- and DNase I-bound Conformer 1**<br>EMD-50516<br>PDB 9FJU	**β/γ-actin pointed end, phalloidin- and DNase I-bound Conformer 2**<br>EMD-50517<br>PDB 9FJY
**Data collection and processing**		
Magnification	81,000	81,000
Voltage (kV)	300	300
Electron exposure (e–/Å^2)	64.6	64.6
Nominal defocus range (μm)	-1.2 to -2.5	-1.2 to -2.5
Pixel size (Å)	0.88	0.88
Symmetry imposed	C1	C1
Initial particle images (no.)	4,418,492	4,418,492
Final particle images (no.)	161,657	178,346
Map resolution (Å)		
0.143 FSC threshold	3.84	3.79
Map resolution range (Å)	3.4 – 6.0	3.4 – 6.0
**Refinement**		
Initial model used (PDB code)	6T20 and 2A42	6T20 and 2A42
Model resolution (Å)		
0.5 FSC threshold	4.1	4.0
Map sharpening *B* factor (Å^2)	-135	-125
Model composition		
Non-hydrogen atoms	15,968	15,968
Protein residues	2,010	2,010
Waters	-	-
Ligands	24	24
B factors (Å^2)		
Protein	104.34	94.50
Waters	-	-
Ligand	105.14	91.98
R.m.s. deviations		
Bond lengths (Å)	0.002	0.002
Bond angles (°)	0.581	0.574
Validation		
EM-ringer score	1.83	2.23
MolProbity score	1.55	1.50
Clashscore	9.90	9.49
Rotamer outliers (%)	0.18	0.35
Ramachandran plot		
Favored (%)	97.85	98.26
Allowed (%)	2.15	1.74
Disallowed (%)	0.00	0.00
