# Supplementary material for: Phalloidin and DNase I-bound F-actin pointed end structures reveal principles of filament stabilization and disassembly
Source: Nat Commun. 2024 Sep 11;15:7969. doi: 10.1038/s41467-024-52251-3 (PMC11390976; doi:10.1038/s41467-024-52251-3)
Supplement: Supplementary file 3 — Description of Additional Supplementary Files [file 41467_2024_52251_MOESM3_ESM.pdf]

Description of Additional Supplementary Files

Supplementary Movie 1. Cryo-EM structures of the F-actin undecorated and phalloidin-bound pointed end.

Supplementary Movie 2. Morph between the F-actin undecorated pointed end and the phalloidin-bound pointed end.

Supplementary Movie 3. Cryo-EM structures of the DNase I- and phalloidin-bound F-actin pointed end.

Supplementary Movie 4. 3D variability of the DNase I- and phalloidin-bound F-actin pointed end.

Supplementary Movie 5. Model of DNase I-mediated disassembly of actin filaments.
