# Supplementary material for: Phalloidin and DNase I-bound F-actin pointed end structures reveal principles of filament stabilization and disassembly
Source: Nat Commun. 2024 Sep 11;15:7969. doi: 10.1038/s41467-024-52251-3 (PMC11390976; doi:10.1038/s41467-024-52251-3)
Supplement: Supplementary file 9 — Reporting Summary [file 41467_2024_52251_MOESM9_ESM.pdf]

nature portfolio

Corresponding author(s): Peter Bieling, Stefan Raunser

Last updated by author(s): August 19th, 2024

Reporting Summary

Nature Portfolio wishes to improve the reproducibility of the work that we publish. This form provides structure for consistency and transparency in reporting. For further information on Nature Portfolio policies, see our Editorial Policies and the Editorial Policy Checklist.

Statistics

For all statistical analyses, confirm that the following items are present in the figure legend, table legend, main text, or Methods section.

n/a	Confirmed	
☐	☒	The exact sample size (*n*) for each experimental group/condition, given as a discrete number and unit of measurement
☐	☒	A statement on whether measurements were taken from distinct samples or whether the same sample was measured repeatedly
☒	☐	The statistical test(s) used AND whether they are one- or two-sided<br>*Only common tests should be described solely by name; describe more complex techniques in the Methods section.*
☒	☐	A description of all covariates tested
☒	☐	A description of any assumptions or corrections, such as tests of normality and adjustment for multiple comparisons
☐	☒	A full description of the statistical parameters including central tendency (e.g. means) or other basic estimates (e.g. regression coefficient) AND variation (e.g. standard deviation) or associated estimates of uncertainty (e.g. confidence intervals)
☒	☐	For null hypothesis testing, the test statistic (e.g. *F*, *t*, *r*) with confidence intervals, effect sizes, degrees of freedom and *P* value noted<br>*Give P values as exact values whenever suitable.*
☒	☐	For Bayesian analysis, information on the choice of priors and Markov chain Monte Carlo settings
☒	☐	For hierarchical and complex designs, identification of the appropriate level for tests and full reporting of outcomes
☒	☐	Estimates of effect sizes (e.g. Cohen's *d*, Pearson's *r*), indicating how they were calculated

Our web collection on statistics for biologists contains articles on many of the points above.

Software and code

Policy information about availability of computer code

Data collection	Cryo-EM data was collected using the commercially available software EPU version 2.8 (Thermofisher Scientific).
Data analysis	Cryo-EM data was screened and collected using EPU v3.7.0.6930. Cryo-EM data collection was monitored and preprocessed on the fly using TranSPHIRE version 1.5.13. The preprocessing steps in TranSPHIRE involved gain and drift correction using UCSF MotionCor2 v1.3.0, CTF estimation with CTFFIND4 v4.1.13, and particle picking using SPHIRE-crYOLO v1.5.8 and v1.8. All cryo-EM data were further processed using RELION v3.1.0 and CryoSPARC v3.3.2 to v 4.2.1. Protein model building was performed in COOT v0.9.8.1 and the models were refined using phenix real-space refine v1.21rc1_5015. Protein models were validated within the phenix suite v1.21rc1_5015. Figures and videos that depict cryo-EM density maps and protein structures were prepared using UCSF ChimeraX v1.5 and v1.6.1.

For manuscripts utilizing custom algorithms or software that are central to the research but not yet described in published literature, software must be made available to editors and reviewers. We strongly encourage code deposition in a community repository (e.g. GitHub). See the Nature Portfolio guidelines for submitting code & software for further information.

Data

Policy information about availability of data

All manuscripts must include a data availability statement. This statement should provide the following information, where applicable:

- Accession codes, unique identifiers, or web links for publicly available datasets
- A description of any restrictions on data availability
- For clinical datasets or third party data, please ensure that the statement adheres to our policy

The cryo-EM maps generated in this study will be deposited in the Electron Microscopy Data Bank (EMDB) under accession codes (dataset in brackets): EMD-50507 (F-actin pointed end), EMD-50506 (phalloidin-bound F-actin pointed end), EMD-50516 (phalloidin- and DNase I-bound F-actin pointed end, maps before 3D classification and conformer 1), EMD-50517 (phalloidin- and DNase I-bound F-actin pointed end, conformer 2). Sharpened and unsharpened maps, unfiltered half-maps and the masks used for refinements are included in each entry. Associated protein models have been deposited in the Protein Data Bank (PDB) with accession codes 9FJO (F-actin pointed end), 9FJM (phalloidin-bound F-actin pointed end), 9FJU (phalloidin- and DNase I-bound F-actin pointed end, conformer 1), 9FJY (phalloidin- and DNase I-bound F-actin pointed end, conformer 2). All other materials are available from the corresponding authors upon request.

Research involving human participants, their data, or biological material

Policy information about studies with human participants or human data. See also policy information about sex, gender (identity/presentation), and sexual orientation and race, ethnicity and racism.

Reporting on sex and gender	n/a
Reporting on race, ethnicity, or other socially relevant groupings	n/a
Population characteristics	n/a
Recruitment	n/a
Ethics oversight	n/a

Note that full information on the approval of the study protocol must also be provided in the manuscript.

Field-specific reporting

Please select the one below that is the best fit for your research. If you are not sure, read the appropriate sections before making your selection.

☒ Life sciences ☐ Behavioural & social sciences ☐ Ecological, evolutionary & environmental sciences

For a reference copy of the document with all sections, see nature.com/documents/nr-reporting-summary-flat.pdf

Life sciences study design

All studies must disclose on these points even when the disclosure is negative.

Sample size	Sample sizes for the three cryo-EM datasets presented in this study: For the pointed end dataset, 20,305 micrographs were collected. 1,935,707 total particles were picked and 206,373 particles were used for the final reconstruction. For the phalloidin-bound pointed end dataset, 20,393 micrographs were collected. 5,200,600 total particles were picked and 280,802 particles were used for the final reconstruction. For the DNase I- and phalloidin-bound dataset, 15,978 micrographs were collected. 4,418,492 total particles were picked and 161,657 (conformer 1) and 178,346 (conformer 2) particles were used for the final reconstructions.<br>These sample sizes of ~20,000 micrographs are common in the cryo-EM field for obtaining high-resolution protein structures from single particle datasets where the particle of interest is not very abundant, see for example Oosterheert, Boiero Sanders et al. Science (2024): https://www.science.org/doi/10.1126/science.adn9560.
Data exclusions	During the cryo-EM image processing, particles that represented false picks or particles that did not contribute high-resolution information to the reconstructions were discarded through 2D and 3D classification procedures. This process, which is required to obtain high-resolution reconstructions, is a standard procedure in cryo-EM image processing.
Replication	The structures of the pointed end, either undecorated of bound by DNase I were observed in previous test datasets at lower resolution. All attempts yielded structures with the same features. The different conformers for the DNase I-bound pointed end were only analyzed at high resolution. All cryo-EM datasets that yielded high-resolution structures for this article were collected in one session per structure and were not repeated. It is unattainable from a time and cost perspective to repeat cryo-EM data collection and processing on the exact same sample.
Randomization	For the 3D refinement of cryo-EM structures, particles were randomly split into two half sets.
Blinding	This study does not involve any experiments where blinding would be applicable, because there were no confounding factors in our experiments that could have led to biased results.

Reporting for specific materials, systems and methods

We require information from authors about some types of materials, experimental systems and methods used in many studies. Here, indicate whether each material, system or method listed is relevant to your study. If you are not sure if a list item applies to your research, read the appropriate section before selecting a response.

Materials & experimental systems

n/a	Involved in the study
☒	☐ Antibodies
☐	☒ Eukaryotic cell lines
☒	☐ Palaeontology and archaeology
☒	☐ Animals and other organisms
☒	☐ Clinical data
☒	☐ Dual use research of concern
☒	☐ Plants

Methods

n/a	Involved in the study
☒	☐ ChIP-seq
☒	☐ Flow cytometry
☒	☐ MRI-based neuroimaging

Eukaryotic cell lines

Policy information about cell lines and Sex and Gender in Research

Cell line source(s)	BTI-Tnao38, species of origin - Trichoplusia ni. Provider: A. Musacchio, MPI Dortmund.<br>Research Resource Identifier: CVCL_Z252.
Authentication	The BTI-Tnao38 cell line was not authenticated.
Mycoplasma contamination	The BTI-Tnao38 cell line was not tested for mycoplasma contamination.
Commonly misidentified lines<br>(See ICLAC register)	The cell line used is not a commonly misidentified line.

Plants

Seed stocks	n/a
Novel plant genotypes	n/a
Authentication	n/a
